# 22 months follow-up of deep marginal acquisition with thermacut bur in management of deep subgingival interproximal carious lesions: a case report

**DOI:** 10.1186/s12903-024-05326-y

**Published:** 2025-01-15

**Authors:** Ahmed Khairy Elmorsy, Shereen Hafez Ibrahim, Hani Mohamed Essam El-Nahass, Ahmed El Zohairy

**Affiliations:** 1 Faculty of Dentistry, Cairo University, B.D.S, Giza, Egypt; 2 Professor of Conservative Dentistry, Faculty of Dentistry, Cairo University, Giza, Egypt; 3 Professor of Periodontology, Faculty of Dentistry, Cairo University, Giza, Egypt

**Keywords:** Deep marginal acquisition, Thermacut bur, Deep subgingival interproximal carious lesions

## Abstract

**Background:**

Minimally invasive dentistry is now becoming the forefront of restorative dentistry, involving less traumatic treatment protocols, conservation of tooth structure and surrounding tissues, enhancing the long-term survivability of treated teeth, and improving the overall quality of life for patients.

**Objective:**

The current case report was conducted to evaluate acquiring deep subgingival interproximal carious lesions by the mean of thermacut bur gingivectomy, in terms of patient satisfaction through pain evaluation, Bleeding on Probing, Pocket Depth, Crestal Bone Level evaluation, and restoration evaluation using modified USPHS criteria.

**Material and methods:**

A patient with a deep proximal cavity in the posterior tooth was thoroughly examined and underwent Thermacut Bur Gingivectomy (TBG) after caries removal followed by direct resin composite restoration of the prepared cavity. Patient Satisfaction using a Visual Analogue Scale (VAS) as a primary outcome. Bleeding on Probing (BoP), Probing Depth (PD), and Crestal Bone Level (CBL) as secondary outcomes, and Marginal Integrity using Modified USPHS Criteria as a tertiary outcome, were evaluated at the baseline, immediate post-operative, 6 month, 12 month and 22 month follow-up intervals.

**Results:**

Thermacut bur gingivectomy revealed minimal immediate post-operative pain, minimal time-consuming procedure, minimal (BoP), appropriate (PD) and no need for extra specialty involvement in the treatment of deep interproximal carious lesions in addition to preservation of the alveolar bone crest with excellent restoration margin at different time intervals.

**Conclusions:**

Thermacut bur gingivectomy can be considered a valid treatment for managing of deep subgingival interproximal carious lesions in vital teeth. Thermacut bur gingivectomy can be introduced as an easy technique for clinicians in the management of deep subgingival interproximal carious lesions, without the need to refer patients to periodontists and without the need for special devices.

## Introduction

Dental restorations play a critical role in maintaining periodontal health, which is critical for the long-term success of interproximal dental restorations [1]. Deep subgingival interproximal carious lesions can create great challenges during restorative procedures using composite restorations, complicating rubber-dam placement by preventing proper inversion and exposing cavity gingival margin, which causes leakage, which in turn can complicate matrix adaptation, adhesion procedure, and composite placement. exposing cavity margins is critical to achieve proper restorative procedure [2].

Now at the forefront of restorative dentistry, minimally invasive dentistry involves less traumatic treatment protocols, preserves the surrounding tissues and tooth structure, increases the long-term survival of treated teeth, and improves patients' overall quality of life. It might be difficult at times to incorporate this idea into the clinical workflow since restorative dentists may have difficulties with deep subgingival interproximal carious lesions, for example. In these situations, functional crown lengthening was thought to be the best course of action [3, 4].

The biological width is a commonly used term used to describe the junctional epithelium and supracrestal connective tissue attachment. This term is now replaced by supracrestal connective tissue attachment. It forms an important tight seal around the tooth, which is critical in protecting the periodontium from any microbial injury and maintaining periodontal health. Infringement of this important landmark, which can happen during restorative treatment, can lead to gingival inflammation, crestal bone resorption, and gingival recession [3].

However—in many clinical scenarios—subgingival carious lesions and crown-root fractures may affect the biologic width dimension. Functional crown lengthening is a surgical procedure that is used to restore this important landmark, which plays an important role in maintaining periodontal health and the long-term stability of restorations. Functional crown lengthening is an effective procedure done before the prosthetic procedure with great success, provided that a certain protocol is being undertaken. It is done by achieving at least a 3 mm distance between the alveolar bone crest and the flap margin at the time of suturing [4]. Its drawbacks include the possibility of root exposure, involvement of the furcation in posterior teeth with high furcation, compromise of the crown-root ratio, risk of implant thread exposure if it is performed in addition to the implant, as well as the extension of crestal bone recontouring to the buccal and lingual walls and, in certain cases, to adjacent teeth in order to achieve smooth bony architecture. Additionally, surgical complications include post-operative pain, inflammation, edema, and the possibility of excessive bleeding [4]. Deep marginal acquisition and deep marginal elevation (DMA & DME) were introduced as a new protocol for managing deep subgingival margins [2, 5], by using a circumferential matrix to acquire deep margins under rubber-dam isolation. However, this technique was intended for cavity design optimization to receive indirect restoration.Other techniques to acquire deep margins have been suggested, like the use of diode laser, electrosurgery, and soft tissue bur [6], however, there is a new protocol for exposing deep subgingival margins through the use of a thermacut bur, which is a bur mounted on a high-speed contra-angle handpiece with no abrasives to ensure the cutting of the papilla and exposing the margin without the risk of damaging tooth surfaces. This technique holds a significant advantage over electrosurgery and diode lasers, in its extremely low cost and no need for special devices or equipment that needs special training.This method is currently used by many clinicians with good follow-ups. However, it has never been tested in research before. which forms a gap in the literature between research and clinical workflow. In this specific incident, the clinicians have leaped forward and introduced a technique. Which should be validated through proper research. The research question suggested was that “Can deep marginal acquisition by means of thermacut bur effective in dealing with deep subgingival margins?” So, this study was designed to assess deep marginal acquisition by the mean of thermacut bur in dealing with deep subgingival interproximal carious lesions to test the hypothesis that thermacut bur is effective minimal invasive approach in deep Margin Acquisition for the management of deep subgingival interproximal carious lesions.

## Materials and methods

### Case presentation

This case report has been described according to the 2013 CARE checklist for case report writing and publishing guidelines [7].

### Materials and armamentarium information

The materials used in this study were as follows: Uni-Etch tooth conditioner gel (Bisco, USA), All bond universal adhesive (Bisco, USA), 3 M Filtek one bulk-fill composite (3 M ESPE, Germany), Capo Flowable Bulkfill Composite (Shuetz Dental). All the Materials’ specifications, composition, LOT numbers, and manufacturers are presented in Table 1. Thermacut bur size 010 (Dentsply, Germany) which is a long shaft smooth tungesten carbide bur (25 mm) with rounded end with no abrasives was used to prevent tooth injury and limit the removal to only gingival tissue.

### Patient information

A 38-year-old female patient visited the conservative dentistry department outpatient clinic, Cairo University, complaining from the presence of interproximal carious lesion that annoyed him as result of frequent food impaction and mild pain.

### Clinical findings

Upon careful preoperative examination procedure, it was found that the patient suffers from deep sub-gingival interproximal carious lesions. Marginal acquisition using pre-wedging wasn’t feasible due to the subgingival location of the cavity margin. Upon radiographic examination, the crestal bone level was less than 2 mm distance (1.9 mm), and after cavity preparation, the distance became 1.8 mm. This distance meant that there is a violation in the biological width. Diagnostic charts containing medical and dental histories were obtained from the patient. All clinical procedures were performed by one expert operator.

### Diagnosis and assessment

**Fig. 1:**
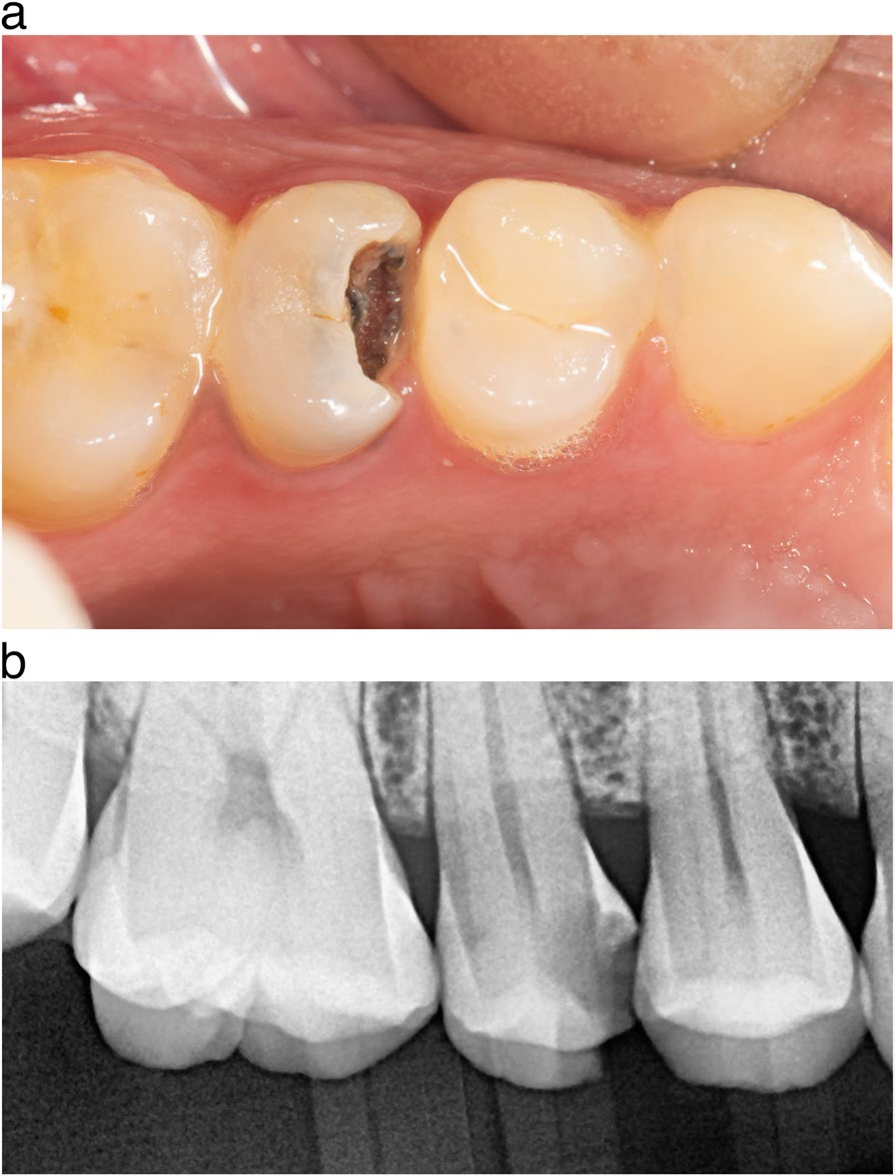
**a** Preoperative occlusal view showing deep subgingival interproximal carious lesion. **b** Preoperative radiograph showing deep subgingival interproximal carious lesion

Since carious lesions are associated with plaque deposits, the dental plaque has to be removed before the assessment. Dental prophylaxis (scaling and polishing) was performed with a bristle brush and fluoridated prophylaxis paste, preoperative photograph, Fig. 1a,b, Preoperative pocket depth using a graded periodontal probe, and pre-operative bitewing radiograph was taken for the participant.

### Therapeutic intervention

**Fig. 2:**
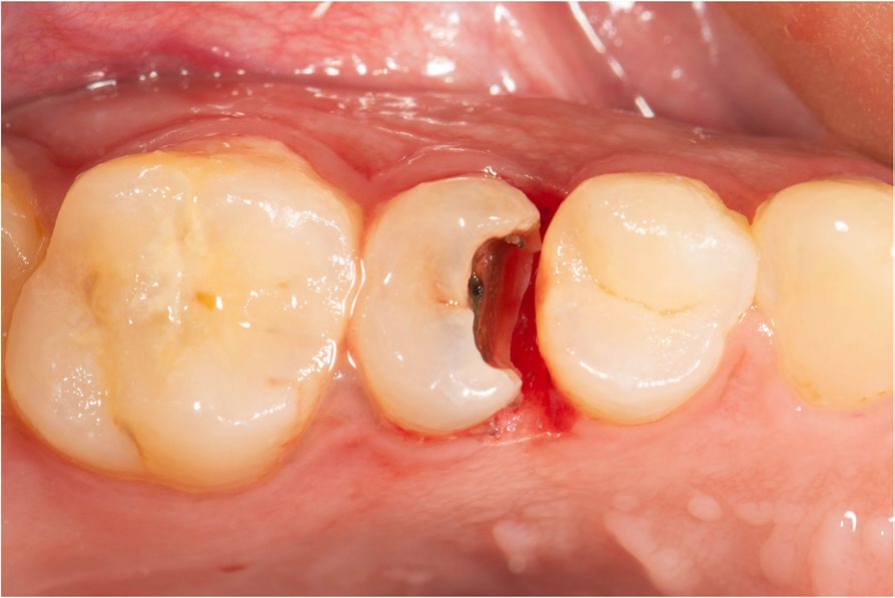
Occlusal view showing papilla removal using thermacut bur

First, local anesthesia (4% articaine with 1:100,000 epinephrine, ArtPharma, Egypt) was given to create field anesthesia using 1.4 ml, then the remaining 0.3 ml was administered to the papilla from both buccal and palatal or lingual aspects to reduce pain and bleeding during papilla removal [6]. Then a thermacut bur mounted on a high-speed 1:5 contra-angle handpiece (Joy Dental, China) was used to cut the papilla at a 90 degree angle and without coolant to acquire deep subgingival margin [6], and to convert the location of the gingival seat from subgingival to a supragingival location extending papilla cutting buccally and lingually to facilitate rubber-dam inversion Fig. 2.

**Fig. 3:**
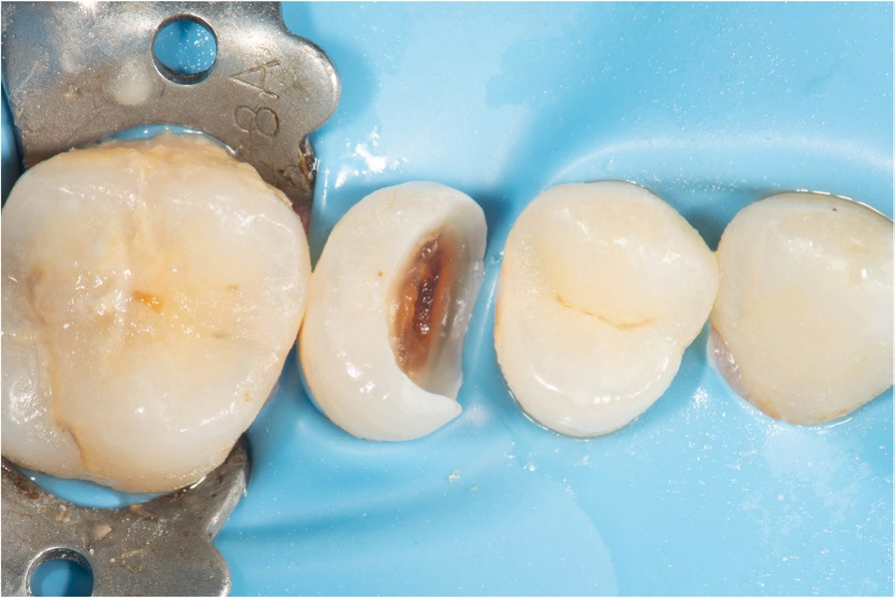
Occlusal view showing rubberdam inversion beneath the cavity margin and partial carious lesion removal

The rubber dam was used to obtain isolation of the operative field. “6X6’’ heavy blue sanctuary rubber dam sheet (Sanctuary, Perak, Malaysia) was applied. The dam and frame were carried to the patient’s mouth first, then the clamp was placed to stabilize the dam at the tooth distal to the tooth to be restored. Then, interproximal placement of the rubber dam sheet and inversion of the dam margins into the gingival sulcus was achieved using dental floss Fig. 3.

Proximal box cavity preparation was performed in all cases by the mean of round diamond burs sizes (009,012, 021) (Komet Dental, Lemgo, Germany) mounted on a 1:5 contra-angle handpiece with 4-way water spray coolant (Joy Dental, China) at 200,000 RPM. The size of the diamond bur was chosen based on carious lesion depth and extension to clean Enamel and DEJ, accessing the proximal cavity from an occlusal direction through the triangular fossa.

**Fig. 4:**
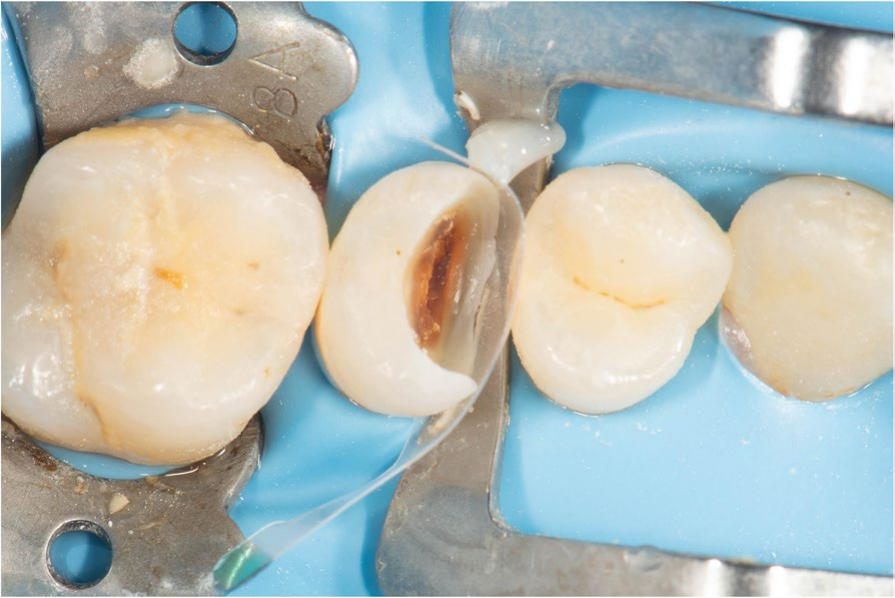
Occlusal view showing sectional matrix and Elliot separator Placement

The cavity outline was driven by carious lesion extension, followed by sharp excavators (Dentsply, Konstanz, Germany), allowing for accessible removal of remaining soft carious lesions using scrapping motion instead of scooping action to avoid pulpal exposure. Cleaning the cavity was done using Aquacare air abrasion device (Velopex, UK) by utilizing (29 um) particle-sized aluminum oxide powder, followed by washing for 15 s to wash out the excess powder. A transparent contoured sectional matrix (Tor Vm, Russia) was placed. The Elliot separator was placed afterward to adapt the matrix cervically and to compensate for the matrix thickness to attain contact tightness Fig. 4.

Acid-etch gel was only applied to enamel (selective enamel etching technique) using a syringe. It was placed for 15–20 s, then rinsed using air and water stream for 20 s, then air dryness until the enamel had a chalky white appearance. After that, 2 coats of All-bond universal adhesive were applied to the enamel and dentin using a micro brush till completely wetting the surface. Vigorous rubbing and agitation were done for 20 s for each coat, followed by air plotting using a gentle stream of air until a uniform layer was formed. Then light curing for 20 s using Elipar S10 (3 M ESPE, USA) light curing unit.

As this case was considered an extremely deep case, delayed wedging was performed by building up a 1.5 mm cervical hip using flowable Bulkfill composite before the Elliot separator was placed. It was done to prevent collapsing the matrix contour cervically or creating an under hang in the restoration, ensuring the matrix was properly self-adapted to the cavity margin and, in some cases, adaptation was enhanced using Teflon tape based on the clinical scenario Fig. 4.

**Fig. 5:**
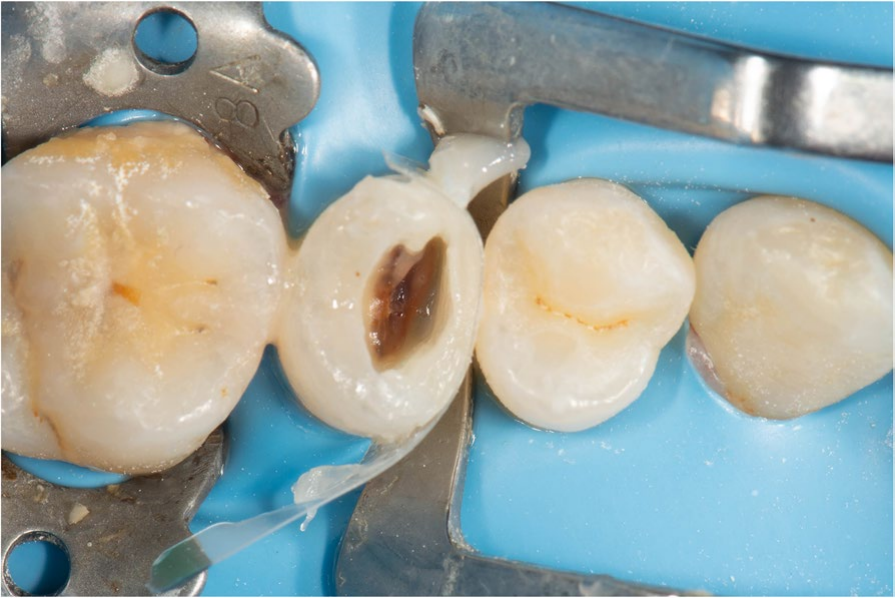
occlusal view showing proximal wall build-up using snow-plow technique

The incremental composite placement was done starting by building up the proximal wall using a combination of flowable and packable composite resin (snow-plow technique). This was to ensure cavity margins were completely covered with composite and decrease the risk of void formation. The choice of Filtek one bulk-fill was to allow curing light to reach the deepest layers of the composite at the very deep margins, and to decrease polymerization shrinkage stresses Fig. 5.

**Fig. 6:**
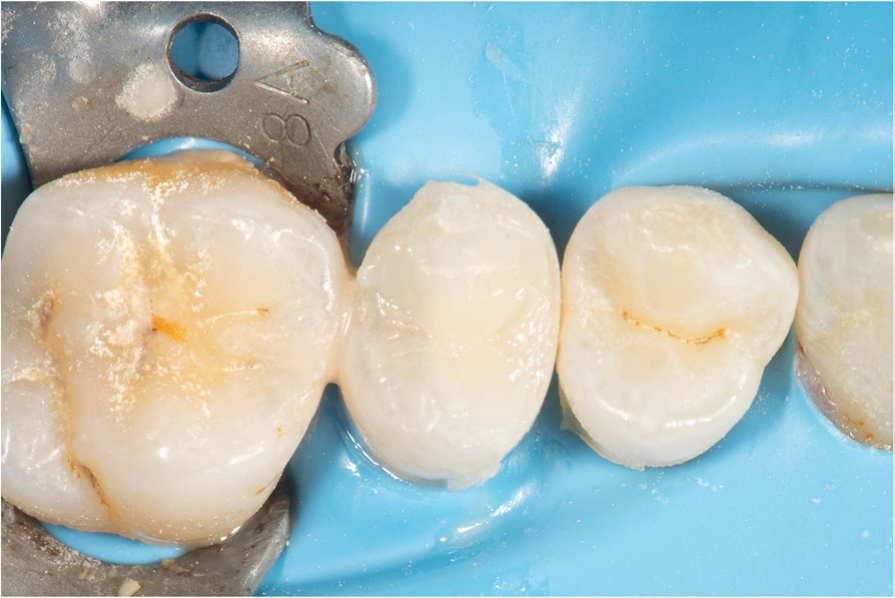
occlusal view showing composite restoration before finishing

The restoration was checked for adaptation to the cavity margins using an explorer, the excess material was removed using Eccesso instrument (LM-ARTE, Parainen, Finland), and the contour was adjusted to minimize the finishing step using yellow coded fine tapered with round end finishing stone (Mani, Tochigi, Japan) Fig. 6.

**Fig. 7:**
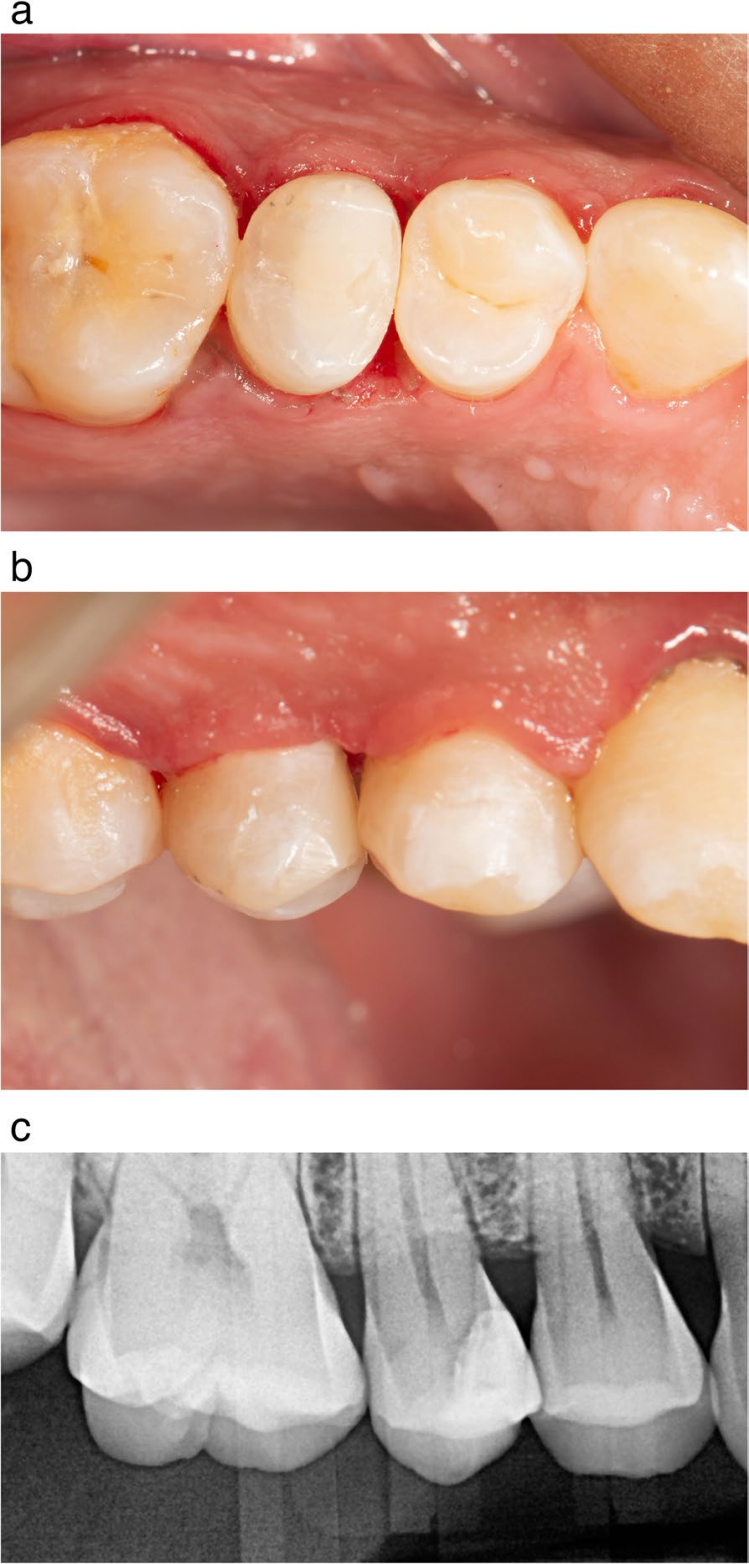
**a** immediate post operative Occlusal view showing composite restoration after finishing and polishing. **b** immediate postoperative buccal view showing the emergence profile of the composite restoration and the removed papilla. **c** immediate postoperative radiograph showing the marginal adaptation of the composite restoration and crestal bone level

Polishing was done using Diacomp Twist polishing rubbers (EVE GmbH, Germany) mounted on a 1:1 low-speed contra-angle handpiece with internal coolant (Kavo, Germany) at 10,000 RPM. An explorer was used to ensure no excess material at the tooth-restorative interface and proper adaptation of the restoration, by moving it from the tooth to the restoration and moving it to the opposite way to ensure no open margins existed. Immediate post-operative bitewing radiograph was taken after completion of restoration to evaluate CBL, Fig. 7a-c, and pocket depth was measured using a graded periodontal probe.

Due to the limitations of this study setting, including small sample size and lack of control group for comparison, the authors highly recommend the need for clinical trials with longer term follow-ups to verify the findings that resulted from this study.

### Case assessment

The current case aimed to assess deep marginal acquisition using thermacut bur gingivectomy in terms of patient satisfaction through pain evaluation using a Visual Analogue Scale as a primary outcome, BoP, PD, and CBL, and association between BoP and CBL as secondary outcomes, and evaluating Marginal Integrity including Marginal Staining Surface Discoloration, and Marginal Adaptation as tertiary outcomes. The assessment was performed by two expert assessors other than the operators (a periodontist and a restorative professors).

#### Primary: patient satisfaction

The patient’s general self‐satisfaction evaluation regarding deep marginal acquisition and functional crown lengthening techniques was performed using a visual analogue scale (VAS) of a 10 cm line with anchors ‘extremely dissatisfied’ at 0 cm and ‘extremely satisfied’ at 10 cm. Higher scores represent greater patient satisfaction with the retraction technique. The subjective nature of this outcome can vary between patients in a larger study settings, that is why other objective methods were used.

#### Secondary outcome

Bleeding on probing (BoP) and Pocket depth (PD) were evaluated before and after deep marginal acquisition using a graded Periodontal Probe. Crestal Bone Level was evaluated using digital radiographs (bitewing with paralleling technique) using a positioning system and fixed using a bite registration material (Regidur from Bisico). Measurement at baseline was done from the cavity margin to the alveolar bone crest, Measurement after completing the restoration was done from the cervical margin to the Alveolar bone crest.

#### Tertiary outcome

Marginal integrity, marginal adaptation, and surface roughness were measured at baseline, immediate post-restorative, 6 month, 12 month, and 22 month using Alpha, Bravo, and Charlie & Delta grading (Table 2).

**Table 1:** Materials & Armamentarium used with details

Material	Specifications	Composition	Manufacturer	LOT#
*Uni-Etch*	A surface-conditioning agent used for enamel and/or dentin treatment prior to adhesive application	Uni-Etch tooth conditioner gel consists of water, 32% phosphoric acid, Benzalkonium Chloride (BAC), silicon dioxide, surfactants and blue colorant	Bisco, USA	E-5502EBM
*All bond universal adhesive*	One component universal adhesive that can be used with. It is characterized by compatible with ALL cements, no post-operative sensitivity	All bond universal adhesive consists of 10-MDP, ethanol, BIS-GMA, HEMA, water, initiator and stabilizer	Bisco, USA	2,400,000,216
*Filtek one Bulkfill Posterior composite*	A radiopaque, light curable composite restorative material used for posterior restorations	AUDMA, UDMA and 1, 12-dodecane-DMA Composite Filler: zirconia/silica cluster filler (comprised of 20 nm silica and 4 to 11 nm zirconia particles) and ytterbium trifluoride filler consisting of agglomerate 100 nm particles. Filler loading: 76.5% wt. % (58.4% vol.)	3 M, Germany	053M4863A2
*Capo Flowable Bulkfill* *Composite*	a light-curing posterior composite resin for the direct filling therapy and for restorations using Bulk Filling. It is suitable for layering with a thickness of up to 4 mm.	Glass powder, aliphatic urethane dimethacrylate, tetramethylene di-methacrylate, silicon dioxide Total filler: 77% by weight (57% by volume) inorganic filler (0.005 – 40 μm)	Shuetz Dental	2,022,001,873

**Table 2:** Tertiary outcome measurements

Clinical Characteristics	Marginal Adaptation	Surface roughness	Marginal staining
Alpha	Explorer does not catch or has one-way catch when drawn across the restoration/tooth interface	The surface of the restoration does not have any surface defects	There is no discoloration between the restoration and tooth
Bravo	Explorer does not catch or has one-way falls into crevice when drawn across the restoration/tooth interface	The surface of the restoration has minimal surface defects	There is discoloration on less than half of the circumferential margin
Charlie	Dentin or base is exposed along the margin	The surface of the restoration has severe surface defects	There is discoloration on more than half of the circumferential margin
Delta	Debonding of the restoration	N/A	N/A

#### Follow-Up and Outcomes

**Fig. 8:**
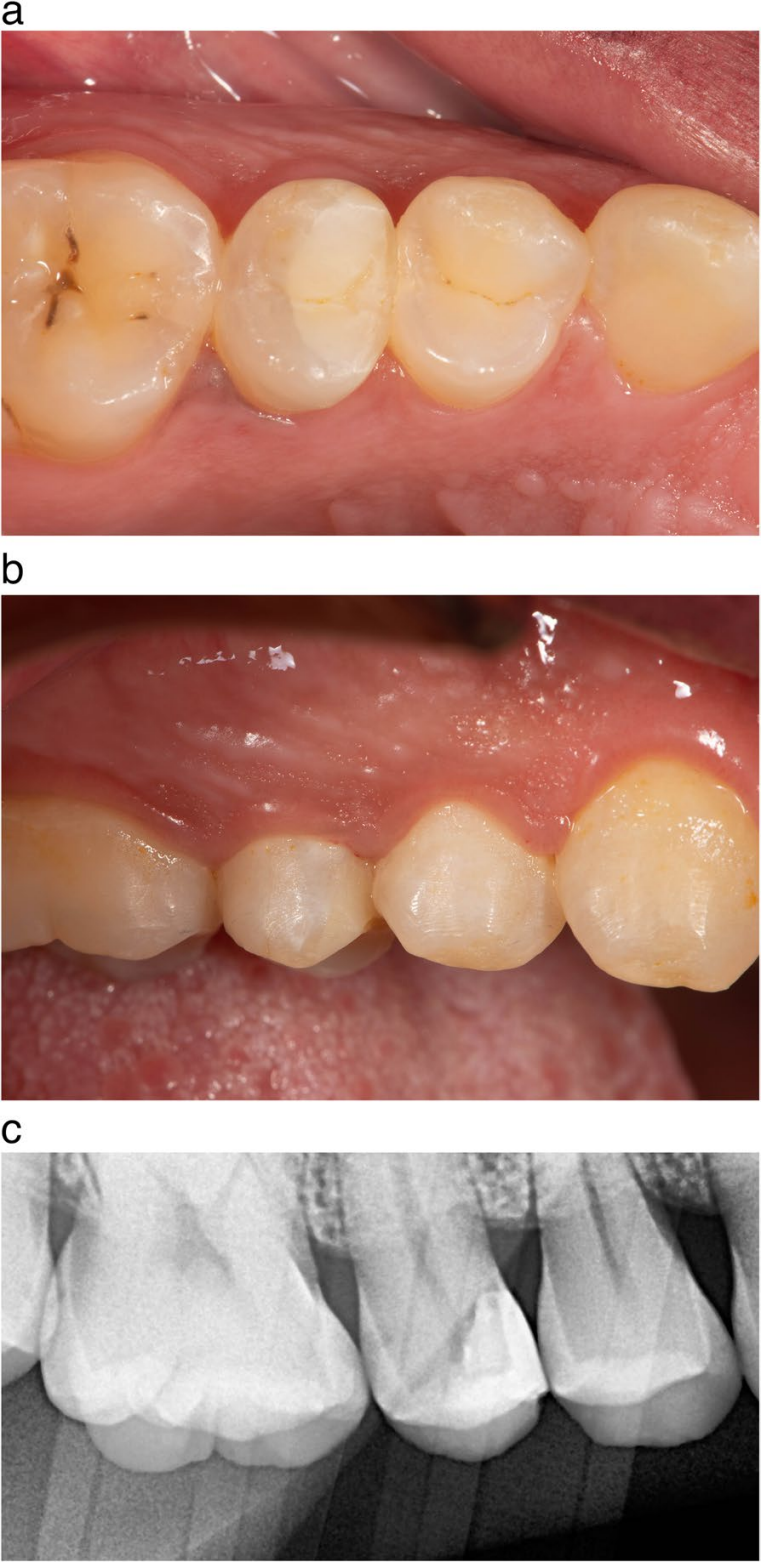
**a** 22-months follow-up occlusal view of the composite restoration. the papilla was completely healed. **b** 22-months follow up buccal view showing papilla complete healing. **c** 22-months follow up radiograph showing marginal adaptation of resin composite restoration and crestal bone level

Directly postoperatively, the patient was presented with a visual analogue scale (VAS) card designed for patient satisfaction with the restorative treatment and she reported the highest level of satisfaction with the provided treatment. Regarding patient satisfaction, immediately post-operative pain, the patient recorded mild pain (score 1) that last for 3 days. However, starting from 6 months and other time intervals, the VAS scale recorded had a zero score. Regarding bleeding on probing, pre-operatively and immediately post-operatively, the patient had bleeding on probing. While no BOP was observed after 6 months and up to 22 months follow up. Regarding pocket depth, the values measured immediately postoperative being lower than those of other intervals (preoperative PD = 3, immediate postoperative PD = 0, after 6 month, 12 months and 22 months PD = 2). Regarding crestal bone level no changes was observed among different time intervals (the score recorded was 1.8 mm). Regarding evaluation of the marginal Integrity (in terms of marginal staining and marginal adaptation) and surface roughness of the restoration using modified USPHS criteria, the restoration had an alpha score during all follow-up intervals Table 3 and Fig. 8a-c.

**Table 3:** Outcome records

outcome	Primary	secondary			tertiary		
Time intervals	Patient Satisfaction (VAS)	(BoP) (Yes/No)	(PD)	Crestal bone level	Marginal integrity	marginal adaptation	surface roughness
immediate post-restorative	Score 1	Yes	0	1.8 mm	alpha	alpha	alpha
6 months	Score 0	No	2	1.8 mm	alpha	alpha	alpha
12 months	Score 0	No	2	1.8 mm	alpha	alpha	alpha
22 months	Score 0	No	2	1.8 mm	alpha	alpha	alpha

## Discussion

Deep subgingival interproximal carious lesions can seriously affect direct restorative workflow, complicating cavity preparation, isolation, matrix placement, separation, and adhesion procedure of the direct restoration. Three decades ago, (Dietschi D and Spreafico R., 1998) introduced Cervical Margin Relocation which was renamed by (Magne and Spreafico, 2012) into Deep Margin Elevation [2]. However, this technique was intended for cavity design optimization (CDO) by modifying the remaining tooth structure to receive indirect restoration, the decision was to use direct composite restoration rather than an indirect approach in the current study [8].

TBG was chosen as an intervention because it is a novel technique, with no previous studies to test its effect in terms of gingival and periodontal health. However, many dentists used it and claimed good follow-ups.

In the current case report, to ensure optimum direct esthetic restorations in those challenging cases, some crucial steps were followed. The marginal acquisition was achieved by doing a gingivectomy using thermacut bur. Then initial cavity preparations were done before rubber dam placement to invert the rubber dam easily below the -caries-free- cavity margins, which became located in a supragingival location concerning gingival level. Another advantage that can be mentioned is to facilitate rubber dam placement by breaking the contact area first, reducing the risk of rubber dam tearing during placement, followed by rubber dam placement and inversion using dental floss. Cavity preparation was finalized by performing partial carious lesion removal to avoid risking traumatic pulp exposure, which may lead to losing vitality or increasing pulpal symptoms without decreasing failure rate when compared to complete carious lesion removal [9].

After cavity preparation and just before the matrix placement step, air particle abrasion to enamel margins was performed using aluminum oxide powder (29 um particle size) to clean the cavity from any residual plaque that may retain on the enamel surface after scaling and polishing procedure and to increase surface area for bonding. Air particle abrasion was performed before enamel etching to maximize bonding potentiality [10].

Sectional pre-contoured anatomical matrices were used in the current study to give the shape and foundation for the proximal walls, matrix height selection was done according to cavity depth and the level of the adjacent marginal ridge, matrix height shouldn’t exceed more than 1 mm of the adjacent marginal ridge. Matrix contour selection was performed based on the distance of the gingival seat related to the adjacent marginal ridge [11]. For matrix cervical adaptation and separation, the Elliot separator was used. It gives a great separation potentiality and allows the composite to flow over the cavity margins to ensure no voids are present in the cavity margins [12].

Selective enamel etching by the mean of a 32% orthophosphoric acid for 15 to 20 s, followed by rinsing for 20 s, to ensure no remnants were found inside the cavity [13]. Proper dryness was performed using an airway syringe to ensure the cavity was dry [14, 15]. Two coats of self-etch adhesive were then vigorously rubbed and agitated inside the cavity to enhance adhesive infiltration. There was no curing between the two layers [16], followed by light curing for 20 s [17].

As an extensively deep cavity, a 1.5 mm cervical hip using flowable bulk fill composite was built before the Elliot separator was placed. To ensure the matrix was properly self-adapted to cavity margin using magnification and—in some cases- adaptation was enhanced using Teflon tape based on the clinical scenario. This provided a superior result when compared to the R2 Technique by Frese, Wolff, and Staehle, 2014 [18], for 2 main reasons, the first step with flowable bulk fill was done in the presence of a rubber dam isolation, and also the presence of a matrix that blocked the Oxygen inhibited layer, which made the first step much smoother than in R2 technique.

Centripetal layering was performed to restore the cavities using the snow-plow technique to build the proximal wall. This technique was chosen to make sure that the combination of flowable and packable composite resin restoration can flow over the cavity margins, to eliminate any risk of voids [19]. Followed by curing for a full 20 s, then completing the rest of the cavity and shaping the composite to the correct shape to reduce the time needed for occlusal adjustment. After that, finishing and polishing of the restoration was done to attain a smooth surface and reduce the risk of surface discoloration and biofilm accumulation [19].

In the current study, Patient Satisfaction through evaluating overall experience including pain and discomfort was the primary outcome measurement. It was chosen because it is a patient-related outcome, representing an important aspect that is usually overlooked in the literature [20]. Patient-related outcomes are of great importance in clinical practice, where inputs from the patients have a great value in measuring their overall experience. A Visual Analogue Scale (VAS) was used to assess the level of pain.

For patient satisfaction as primary outcome with the restorative treatment and she reported the highest level of satisfaction with the provided treatment. This may be attributed to the simplicity of the procedure of papilla removal using a thermacut bur, and only after anesthesia was injected into the papilla before removal with no flap raising or bone removal nor suturing.

Secondary outcomes including BoP, PD, and CBL were measured using a graded periodontal probe and bitewing radiograph with a paralleling technique, respectively. Regarding bleeding on probing, pre-operatively and immediately post-operatively, the patient had bleeding on probing. While no BoP was observed after 6 months and up to 22 months follow up. Regarding pocket depth, the values measured immediately postoperative being lower than those of other intervals (preoperative PD = 3, immediate postoperative PD = 0, after 6 months, one year and 22 months PD = 2). Regarding CBL, no changes was observed among different time intervals (the score recorded was 1.8).

Positive BoP during the preoperative evaluation can be explained by the presence of cavitation and food impaction, which caused inflammation and subsequent BoP. These results showed a favorable response of the periodontium to the treatment modality. It is also worth mentioning that maintaining good oral hygiene was paramount to achieving these results. These results came into agreement with the work of Oppermann et al., 2016 [20] and Farouk et al., 2024 [21], showing a significant decrease in BoP values after 6 months of follow-up and one year respectively with surgical crown lengthening. However, there was a disagreement with Ferrari et al., 2018 [22], who published a conflicting result concerning BoP, showing an increase in the incidence of BoP after 1-year follow-up that was explained by the presence of surface roughness of the flowable composite used for deep marginal elevation. The presence of overhanging in the flowable composite due to the inability to control the flowable composite placement using a matrix, or failure to remove excess composite material or adhesive flashes. Which might have persisted causing plaque accumulation, and interfered with oral hygiene measures, which can lead to a higher risk of bleeding on probing [23]. Here comes the importance of pre-wedging and proper matricing and finishing of the restoration as performed in the current case report to avoid such complications. As the restoration was performed under magnification to ensure superior restorative procedures were being delivered to the patient, also, patient was instructed to follow strict oral hygiene measures including tooth brushing and flossing to maintain a healthy periodontium.

As for pocket depth the obtained result might be attributed to the removal of the papilla with thermacut bur, which leads to the elimination of the sulcus causing the readings with graded periodontal probe near zero at the immediate post-operative interval with no obvious signs of inflammation, including redness or bleeding on brushing. This also concurs with the work of Frese, Wolff and Staehle, 2014 [18], Venuti P. DDS. and Mirabella Eclano., 2018 [6], and Farouk et al., 2024 [21]. In which there was neither clinical significance in pocket depth, nor patient-related complications.

A very interesting clinical insight that should be mentioned here, is that there was no apparent black triangle beneath contact areas under restorations The reason for that can be due to the presence of crestal bone support under the papilla and the architecture of the alveolar bone crest and because of the preservation of the alveolar bone crest position and architecture, the papilla healed and closed the gap fully under the contact areas. pre-operative values were higher than immediate and in other intervals, which can be explained by the effect of cavity preparation causing the increase in depth of the cavity cervical margin, as a result of carious lesion removal. This indicated that polished, smooth, and non-irritating subgingival margins can prevent any negative impact on periodontal tissues. This came into agreement with the work of Frese, Wolff, and Staehle, 2014 [18] and Farouk et al., 2024 [21], which showed very minimal changes to crestal bone level over 12 months.

The biocompatibility of resin composite materials with the surrounding periodontal tissue depends on many criteria. It includes the chemistry of the polymerizable organic matrix of the resinous material, degree of conversion, type of ceramic fillers, and degree of conversion of the material (Beltrami et al., 2021) [24]. Resin composite materials used for the direct restorative procedure were bulk-fill nano-based materials, which are characterized by reduced free monomers due to a higher degree of conversion [25, 26]. These features resulted in better biocompatibility and better periodontal tissue response.

Regarding evaluation of the marginal Integrity (in terms of marginal staining and marginal adaptation) and surface roughness of the restoration using modified USPHS criteria, the restoration had an alpha score during all follow-up intervals. This could be explained by proper composite restoration placement and curing against the matrix surfaces, which were highly surface polished. Surface smoothness plays an important role in decreasing biofilm attachment to the restoration leading to better healing of the soft tissue around composite restorations. This concurs with the work of Frese, Wolff, and Staehle, 2014 [18] and Samartzi et al., 2022 [27]. Their clinical observations showed that plain, smooth, and non-irritating margins on deep interproximal composite restorations infringing the junctional epithelium could be free of gingival and periodontal inflammation. It should be noted that strict oral hygiene measures were followed.

## Conclusions

Within the limitiation of this study the following conclusions can be drawn, Thermacut bur gingivectomy can be considered as an option for the management of deep subgingival interproximal carious lesions in vital teeth because of reduced immediate post-operative pain, length of the procedure, preservation of crestal bone level and no need for extra specialty involvement in the treatment of deep interproximal carious lesions. However, well structured randomized controlled trials with long term follow-up may be needed.

## Recommendation

Thermacut bur gingivectomy can be introduced as an easy technique for clinicians in the management of deep subgingival interproximal carious lesions, without the need to refer patients to periodontists and without the need for special devices. However, due to the lack of adequate sample size and lack of control group, the authors highly recommend the need for future clinical trials with long term follow ups to verify the findings of this novel and minimally invasive procedure.

## Data Availability

The authors report no conflicts of interest in this work. data will be available upon reasonable request.

## Abbreviations

MDP: 10-Methacryloyloxydecyl dihydrogen phosphate
HEMA: Hydroxy Ethyl Methacrylate
UDMA: Urethane Dimethacrylate resin
TEGDMA: Triethyleneglycol Dimethacrylate
AUDMA: Aliphatic Urethane Dimethacrylate resin
DMA: Dimethacrylate resins
TBG: Thermacut Bur Gingivectomy
DME: Deep Margin Elevation
FCL: Functional Crown Lengthening
BoP: Bleeding on Probing
PD: Probing Depth
CBL: Crestal Bone Level
CDO: Cavity Design Optimization
mm: Millimeters
PBE: Proximal Box Elevation
CEJ: Cemento-Enamel Junction
RE: Root Exposure
UA: Universal Adhesive
VAS: Visual Analogue Scale
CBL: Crestal Bone Level
BAC: Benzalkonium Chloride
